# Characterization of the Phosphoproteome in Human Bronchoalveolar Lavage Fluid

**DOI:** 10.1155/2012/460261

**Published:** 2012-09-11

**Authors:** Francesco Giorgianni, Valentina Mileo, Dominic M. Desiderio, Silvia Catinella, Sarka Beranova-Giorgianni

**Affiliations:** ^1^Department of Pharmaceutical Sciences, The University of Tennessee Health Science Center, Memphis, TN 38163, USA; ^2^Corporate Preclinical R&D, Analytics and Early Formulations Department, Chiesi Farmaceutici S.p.A., 43122 Parma, Italy; ^3^Department of Neurology, The University of Tennessee Health Science Center, Memphis, 38163 TN, USA; ^4^Charles B. Stout Neuroscience Mass Spectrometry Laboratory, The University of Tennessee Health Science Center, Memphis, 38163 TN, USA

## Abstract

Global-scale examination of protein phosphorylation in human biological fluids by phosphoproteomics approaches is an emerging area of research with potential for significant contributions towards discovery of novel biomarkers. In this pilot work, we analyzed the phosphoproteome in human bronchoalveolar lavage fluid (BAL) from nondiseased subjects. The main objectives were to assess the feasibility to probe phosphorylated proteins in human BAL and to obtain the initial catalog of BAL phosphoproteins, including protein identities and exact description of their phosphorylation sites. We used a gel-free bioanalytical workflow that included whole-proteome digestion of depleted BAL proteins, enrichment of phosphopeptides by immobilized metal ion affinity chromatography (IMAC), LC-MS/MS analyses with a linear ion trap mass spectrometer, and searches of a protein sequence database to generate a panel of BAL phosphoproteins and their sites of phosphorylation. Based on sequence-diagnostic MS/MS fragmentation patterns, we identified a collection of 36 phosphopeptides that contained 26 different phosphorylation sites. These phosphopeptides mapped to 21 phosphoproteins including, for example, vimentin, plastin-2, ferritin heavy chain, kininogen-1, and others. The characterized phosphoproteins have diverse characteristics in terms of cellular origin and biological function. To the best of our knowledge, results of this study represent the first description of the human BAL phosphoproteome.

## 1. Introduction 

 Posttranslational modification of proteins by phosphorylation plays a complex and critical role in the regulation of numerous biological processes. In recent years, large efforts have been devoted to global-scale analysis of protein phosphorylation sites using various phosphoproteomics methodologies [1, 2]. These phosphoproteomics studies have focused chiefly on large-scale characterization of the phosphoproteomes in cultured cells or tissues. In contrast, investigation of the phosphoproteomes in biological fluids is an emerging area, and studies of this type are relatively scarce. Characterization of protein phosphorylation in biological fluids presents a major challenge. Phosphoproteins released into the fluid are diluted and mostly of low abundance, and they are present in a highly complex mixture, that is composed predominantly of nonphosphorylated proteins. The issue is often compounded by overabundance of certain proteins such as albumin and immunoglobulins. Highly advanced bioanalytical strategies that have been developed and applied successfully in the context of cell and tissue phosphoproteomics are now being tailored for biological fluid phosphoproteomics. 

Recent studies of phosphoproteomics of biological fluids include serum and plasma [3–5], CSF [6, 7], saliva [8], and urine [9]. In particular, examination of phosphoproteomes in biological fluids obtained from sites proximal to specific organs represents a potential route to important mechanistic information as well as to biomarker discovery.

 Human bronchoalveolar lavage fluid (BAL) is a proximal fluid commonly used for diagnosis of lung diseases including chronic obstructive pulmonary disease (COPD) and lung cancer. Procurement of clinical BAL specimens involves washing of the epithelial lining of the lung with saline using a fiberoptic bronchoscope. Molecular composition of BAL reflects the status of the respiratory tract, and analysis of human BAL composition at the molecular level therefore provides an attractive way towards improved understanding of disease mechanisms or discovery of biomarker signatures that are directly relevant to specific lung diseases. The proteome of human BAL has been studied numerous times in the context of various lung diseases [10–14]. In contrast, the phosphoproteome of human BAL has not been characterized yet. 

 In this study, we undertook a pilot interrogation of the human BAL phosphoproteome. Our ongoing research program focuses on proteomics of human BAL [15], and we aim to expand this program to encompass studies of posttranslational modifications. Initially, we set out to determine if phosphorylated proteins can be characterized in human BAL using a mass spectrometry-based analytical platform, and to obtain a first description of the BAL phosphoproteome, including assignments of the sites of phosphorylation.

## 2. Methods

### 2.1. Characteristics of BAL Specimens

 The human BAL specimens were provided by Chiesi Farmaceutici, Parma Italy; the project was approved by the IRB at The University of Tennessee Health Science Center. The human BAL samples were obtained from subjects without clinical diagnosis of COPD or lung cancer. Information on the characteristics of the BAL specimen donors is listed in Table 1. The lavage was performed with four aliquots of 50 mL of saline delivered via a fiberoptic bronchoscope. After centrifugation, the liquid component of BAL was aliquoted and stored at −80°C until analysis. To provide sufficient amount of protein, pooled BAL samples were used. Two separate pools of 3 (Pool 1) and 7 samples (Pool 2), respectively, were analyzed in two independent experiments. 

### 2.2. Sample Desalting and Protein Depletion

 Prior to analysis, the BAL samples were centrifuged to remove cell debris. Processing of each sample included removal of salts and depletion of overabundant contaminant proteins. Desalting was performed by ultrafiltration with spin concentrators (MW cutoff of 5,000 Da). The samples in the concentrators were centrifuged (25 min; 5,000 g; 4°C) to produce *ca.* 100–200 *μ*L of retentate. After the first concentration step, water (4 mL) was added to the retentate and the concentration step was repeated for a total of three times. The final retentates (*ca*. 100 *μ*L) were dried in a vacuum centrifuge. 

 Albumin and five other high-abundance proteins were removed with the Hu-6 Multiple Affinity Removal System (MARS) spin cartridge (Agilent) following procedure provided by the manufacturer. After MARS depletion, the samples were desalted by ultrafiltration as described above. Protein concentration before and after MARS depletion was determined with the micro BCA assay (Pierce). After pooling, the final protein content was 450 *μ*g (Pool 1) and 900 *μ*g (Pool 2).

### 2.3. Whole Proteome Digestion and IMAC Enrichment

 The proteins were digested with trypsin using an in-solution digestion procedure. Briefly, the dried proteins in each pooled sample were redissolved in 45 *μ*L of 400 mM ammonium bicarbonate buffer containing 8 M urea (pH 8). Prior to digestion, the proteins were reduced with DTT (5 *μ*L of 50 mM solution, incubation for 1 h at 56°C) followed by alkylation with iodoacetamide (5 *μ*L of 200 mM solution, incubation for 45 min at room temperature in the dark). The sample was diluted with water to 2 M final urea concentration, and 20 *μ*g of sequencing-grade trypsin (Promega) were added. The mixture was incubated overnight at 37°C.

After digestion, the mixture was acidified with TFA and subjected to solid phase extraction using a home-made SPE minicolumn packed with C18 stationary phase. After elution from the minicolumn, the sample was dried and the redissolved in 90% water/10% acetic acid, as required for immobilized metal ion affinity chromatography (IMAC).

 The IMAC procedure, which serves to enrich the proteolytic digests for phosphopeptides, was performed with the Phosphopeptide Isolation Kit (gallium/IDA, Pierce). Each BAL peptide digest was applied to the column, and the phosphopeptides were bound by incubation at room temperature for 1 h. The column was washed with the following solutions: 40 *μ*L of 0.1% acetic acid (2 washes), 40 *μ*L of 0.1% acetic acid/10% ACN (2 washes), and 40 *μ*L of water (2 washes). The phosphopeptides were eluted from the IMAC column with two 40 *μ*L-aliquots of 200 mM sodium phosphate (pH 8.4), followed by a single elution with 40 *μ*L of 100 mM sodium phosphate/50% ACN. The eluates were combined, the resulting sample was acidified, and its volume was reduced to ca 25 *μ*L in a vacuum centrifuge. Prior to LC-MS/MS analysis, the IMAC-enriched phosphopeptides were desalted with ZipTipC18 (Millipore, Billerica, MA, USA), using the procedure provided by the manufacturer. The phosphopeptides bound to the ZipTipC18 column were eluted with 4 *μ*L of 50% ACN/0.1% formic acid and diluted with 6 *μ*L of 0.5% formic acid; aliquots of these samples were injected onto the LC-MS/MS instrument.

### 2.4. LC-MS/MS and Phosphoprotein Identification

 The LC-MS/MS analyses were performed with an LTQ linear ion trap mass spectrometer (Thermo Electron) that was interfaced with a nano-LC system (Dionex). The IMAC-enriched peptide digests were loaded onto a fused-silica microcapillary column/spray needle (Picofrit, 15 cm length, 75 *μ*m I.D.; New Objective) packed in-house with C18 stationary phase (Michrom Bioresources). The peptides were separated using a 90-min linear gradient from 0% to 90% mobile phase B. Mobile phase B was 10% water/90% methanol/0.05% formic acid; mobile phase A was 98% water/2% methanol/0.05% formic acid. The LC-MS/MS data were acquired in the data-dependent mode. Each of the pooled samples (Pool 1 and Pool 2) was analyzed in triplicate. 

 The LC-MS/MS datasets were used to search the UniProt database (subset of human proteins) using TurboSEQUEST search engine that was part of Bioworks 3.2 (Thermo Electron). The following parameters were used in the searches: full-trypsin specificity, dynamic modifications of phosphorylated S, T, and Y (+80.0), and dynamic modifications of oxidized M (+16.0). The search results were filtered to include peptides retrieved XCorr values ≥2.00, and 3.50 for doubly and triply charged precursor ions, respectively. All MS/MS spectra for the individual phosphopeptides that passed this initial filtering were inspected manually. This manual validation checked for the presence of a product ion that corresponds to the neutral-loss of phosphoric acid ([M+2H-98]^2+^ for doubly charged ions or [M+3H-98]^3+^ for triply charged ions); and for coverage of the phosphopeptide sequence by the b- and/or y product-ion series. Assignments of the sites of phosphorylation were verified by inspecting the b- and/or y-product ions that flanked the phosphorylation site assigned by the search engine. Data from analyses of Pool 1 and Pool 2 were combined to produce the final phosphoprotein panel. Additional information about the phosphorylation sites/phosphoproteins was obtained from the UniProt annotations, the Phosphosite knowledgebase (http://www.phosphosite.org/), the Human Protein Atlas knowledgebase (http://www.proteinatlas.org/), the Ingenuity Pathway Analysis tool (IPA), and from searches of primary literature.

## 3. Results and Discussion

**Figure 1:**
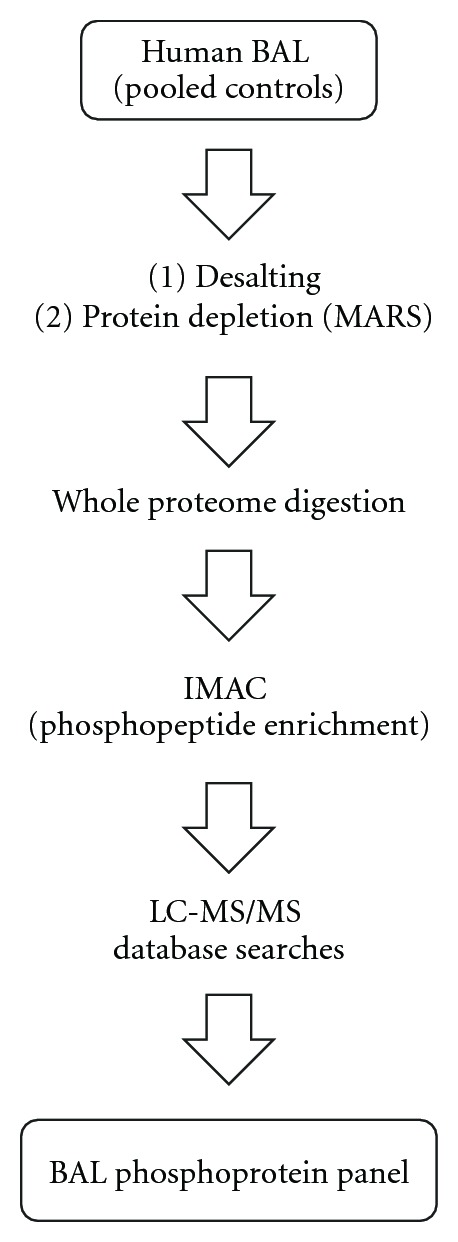
Flowchart depicting the bioanalytical workflow used for BAL phosphoproteome mapping. Abbreviations: Multiple Affinity Removal System—MARS; immobilized metal ion affinity chromatography—IMAC.

**Figure 2:**
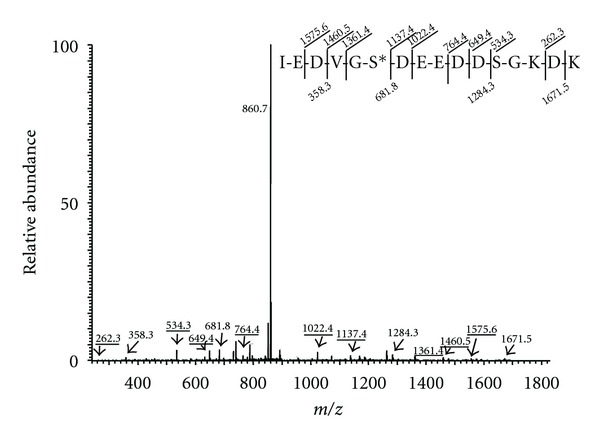
Representative MS/MS spectrum obtained in analyses of IMAC-enriched digests of depleted BAL proteomes. The spectrum displays a prominent product ion at *m/z* 860.7 that corresponds to loss of H_3_PO_4_ from the molecular ion ([M+2H]^2+^ at *m/z* 909.9). Furthermore, y- and b-ions are present that define phosphopeptide sequence and site of phosphorylation. The peptide IEDVGpSDEEDDSGKDK belongs to HSP 90-beta.

 For this pilot study, a simple gel-free bioanalytical strategy was employed. The general outline of the bioanalytical workflow is shown in Figure 1. Specific characteristics of human BAL have to be taken into account for sample processing and protein extraction. First, proteins in BAL are diluted in saline, and therefore sample concentration and desalting are needed. Second, high background created by overabundant plasma proteins would interfere with analysis of low-level phosphoproteins, and removal of these proteins must be accomplished. In our study, to process the BAL samples for phosphoproteome analysis, salts were removed by ultrafiltration, and overabundant plasma proteins were depleted using immunoaffinity capture. Proteins in the depleted BAL samples were digested with trypsin, and the digests were subjected to immobilized metal ion affinity chromatography (IMAC) enrichment for phosphopeptides. The enriched digests were analyzed by LC-MS/MS on an LTQ ion trap mass spectrometer to obtain MS/MS data that indicate the phosphopeptide sequences and phosphosite locations in these peptides. The phosphopeptides and phosphoproteins were identified through searches of the UniProt protein sequence database. Manual inspection of all phosphopeptide search results and of the corresponding MS/MS data was performed to confirm the validity of the phosphopeptide matches. Of diagnostic value in the context of MS/MS fragmentation was the neutral loss of the elements of phosphoric acid from the phosphopeptide molecular ions. This fragmentation pathway, which is prominent in the ion trap mass spectrometer, leads to the appearance of a characteristic product ion in the MS/MS spectrum of a phosphopeptide [16]. This well-known scenario is illustrated in Figure 2, which shows the MS/MS spectrum for the phosphopeptide IEDVGpSDEEDDSGKDK. This spectrum displays a prominent product ion at *m/z* 860.7, which corresponds to the loss of the elements of phosphoric acid from the doubly charged precursor ion. In addition, a number of product ions from the b- and y-series are present that determine the amino acid sequence of the phosphopeptide. Peaks at *m/z* 682 (b_6_) and *m/z* 1137 (y_10_) indicate the exact location of the phosphorylation site on Ser 255 of human heat shock HSP 90-beta. 

Each of the two pooled BAL samples that were analyzed produced a set of 13 phosphoproteins. Five of these phosphoproteins were common to both samples; in addition, each sample yielded a unique group of phosphoproteins. This is not unexpected given the large biological variability associated with clinical specimens, and variable phosphoprotein signatures have been also observed for other clinical samples [17]. The results of our BAL phosphoproteome analyses are summarized in Table 2. Overall, interrogation of the IMAC-enriched digests of depleted BAL samples with LC-MS/MS resulted in the characterization of 36 unique phosphopeptides that contained a total of 26 phosphorylation sites and mapped to 21 proteins. Our results demonstrate that characterization of BAL phosphoproteome is feasible, and the phosphoprotein panel represents new findings that expand our knowledge of the molecular characteristics of BAL proteins. 

Since the focus of our pilot study reported here was on first description of the human BAL phosphoproteome, the scope of the study was limited to qualitative analyses of a small number of specimens from female donors only. This initial examination was not intended to characterize phosphoprotein biomarkers associated with a specific lung disease but to initiate the building of a detailed phosphoprotein/phosphosites catalog as a starting point for future differential phosphoproteomics efforts. In terms of the size of our initial BAL phosphoprotein panel, our results are comparable, for example, to a CSF phosphoproteome study that revealed 44 phosphoproteins [6], or to a recently published catalog of the urine phosphoproteome that included 45 phosphopeptides from 31 proteins [9]. Our initial exploration of the BAL phosphoproteome was not expected to yield a complete description of all BAL phosphoproteins, and it is possible that some phosphoproteins escaped detection due to their low abundance, unfavorable properties of the corresponding phosphopeptides influencing their behavior during analyses, and other issues. Clearly, these pilot results can be expanded in future efforts to enhance the BAL phosphoproteome coverage through modifications of the bioanalytical workflow such as incorporation of additional separation/enrichment dimensions. 

**Figure 3:**
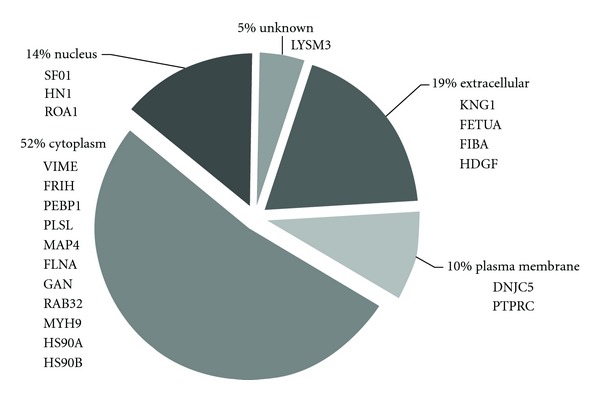
Subcellular location distribution of the characterized proteins; compiled from Ingenuity.

Bronchoalveolar lavage samples components of the epithelial lining fluid, and proteins that are found in BAL are of diverse origin [14]. They may be released by different types of resident and/or infiltrating cells; many plasma proteins are also identified in BAL. To supplement our experimental findings on the phosphorylation status of BAL proteins, we compiled additional information on protein localization from several protein knowledgebases, including Ingenuity and the Human Protein Atlas, HPA (see Table 3 and Figure 3). Regarding tissue-specific protein expression, inspection of protein expression profiles in HPA showed that the majority of proteins from our dataset are expressed in the lung and in other tissues/organs. Analysis of subcellular compartment categories for proteins from our panel showed a strong representation of cytoplasmic proteins (52%); four proteins (19%) were classified as extracellular and include plasma proteins kininogen-1 and alpha-2-HS-glycoprotein whose phosphorylated counterparts have also been characterized in human plasma/serum phosphoproteome [3, 5]. 

The BAL phosphoprotein panel (Table 2) includes proteins with diverse functional characteristics, including structural proteins (vimentin, plastin-2), transcriptional regulators (Splicing factor 1, hepatoma-derived growth factor), chaperones (heats shock protein HSP 90-alpha and -beta), and others. Several of the proteins whose phosphorylation was characterized here have known connection to lung function and perturbations due to environmental stresses including smoking, and/or to lung disease.

 For example, ferritin is an important mediator of iron homeostasis, and increased levels of ferritin have been found in the lavage of smokers [18]. The rationale for this increase is, at least is part, that smoke particles cause iron accumulation in the respiratory tract, and increased expression of ferritin is part of the host response, aimed to sequester the iron. 

 Another phosphoprotein found in the present study is the actin-bundling protein plastin-2. Phosphorylation of plastin-2 modulates its function in the assembly of actin networks, and it is associated with leukocyte activation in response to various stimuli [19, 20]. Recently, plastin-2 has been identified in human BAL proteome as a component of a pulmonary disease marker profile [13].

In conclusion, this study presents novel findings towards description of the human BAL phosphoproteome. Since aberrant protein phosphorylation associated with specific lung diseases could potentially be reflected as alterations in BAL phosphoproteins, this study lays an important foundation for future differential phosphoprotein profiling for biomarker discovery.

## Figures and Tables

**Table 1:** Characteristics of BAL specimen donors.

Donor	Disease status	Gender	Age
1	control	F	48
2	control	F	68
3	control	F	58
4	control	F	75
5	control	F	58
6	control	F	64
7	control	F	63
8	control	F	60
9	control	F	65
10	control	F	73

**Table 2:** Phosphopeptides and phosphoproteins characterized in human BAL.

Database accession code	Entry name	Protein name Phosphopeptide characterized^a^	Site^b^
(1) P08670	VIME_HUMAN	Vimentin	
		QVQS*LTCEVDALK	S325
(2) P02794	FRIH_HUMAN	Ferritin heavy chain	
		KM^#^GAPESGLAEYLFDKHTLGDS*DNES	S179
		KMGAPESGLAEYLFDKHTLGDS*DNES	S179
		MGAPESGLAEYLFDKHTLGDS*DNES	S179
		HTLGDS*DNES	S179
		KMGAPESGLAEYLFDKHTLGDSDNES*	(S183)
		MGAPESGLAEYLFDKHTLGDSDNES*	S183
(3) P30086	PEBP1_HUMAN	Phosphatidylethanolamine-binding protein 1	
		NRPTS*ISWDGLDSGK	S52
(4) P13796	PLSL_HUMAN	Plastin-2	
		GS*VSDEEM^#^M^#^ELR	S5
		GS*VSDEEMM^#^ELR	S5
		GS*VSDEEMMELR	S5
		EGES*LEDLMK	S257
(5) Q9H3Z4	DNJC5_HUMAN	DnaJ homolog subfamily C member 5	
		S*LSTSGESLYHVLGLDK	(S8)
(6) P27816	MAP4_HUMAN	Microtubule-associated protein 4	
		DVT*PPPETEVVLIK	T521
(7) P21333	FLNA_HUMAN	Filamin-A	
		RAPS*VANVGSHCDLSLK	S2152
		CSGPGLS*PGMVR	S1459
(8) P08575	PTPRC_HUMAN	Receptor-type tyrosine-protein phosphatase C	
		NRNS*NVIPYDYNR	S973
(9) P01042	KNG1_HUMAN	Kininogen-1	
		ETTCSKES*NEELTESCETK	S332
(10) P02765	FETUA_HUMAN	Alpha-2-HS-glycoprotein	
		CDSSPDS*AEDVRK	S138
		CDSSPDS*AEDVR	S138
(11) Q15637	SF01_HUMAN	Splicing factor 1	
		TGDLGIPPNPEDRS*PS*PEPIYNSEGK	S80; S82
(12) Q9UK76	HN1_HUMAN	Hematological and neurological expressed 1 protein	
		RNS*SEASSGDFLDLK	(S87)
(13) Q7Z3D4	LYSM3_HUMAN	LysM and putative peptidoglycan-binding domain-containing protein 3	
		S*TSRDRLDDIIVLTK	(S53)
(14) P02671	FIBA_HUMAN	Fibrinogen alpha chain	
		PGSTGTWNPGS*SER	S364
(15) P09651	ROA1_HUMAN	Heterogeneous nuclear ribonucleoprotein A1	
		SES*PKEPEQLR	(S6)
(16) P51858	HDGF_HUMAN	Hepatoma-derived growth factor	
		AGDLLEDS*PKRPK	S165
		RAGDLLEDS*PK	S165
		AGDLLEDS*PK	S165
		GNAEGSS*DEEGKLVIDEPAK	(S133)
(17) Q9H2C0	GAN_HUMAN	Gigaxonin	
		FGAVACGVAMELY*VFGGVR	Y471
(18) Q13637	RAB32_HUMAN	Ras-related protein Rab-32	
		DSS*QSPSQVDQFCK	(S152)
(19) P35579	MYH9_HUMAN	Myosin-9	
		KGAGDGS*DEEVDGK	S1943
(20) P07900	HS90A_HUMAN	Heat shock protein HSP 90-alpha	
		DKEVS*DDEAEEK	S231
(21) P08238	HS90B_HUMAN	Heat shock protein HSP 90-beta	
		IEDVGS*DEEDDSGKDKK	S255
		IEDVGS*DEEDDSGKDK	S255
		IEDVGS*DEEDDSGK	S255

^a^STY* denotes phosphorylated amino acid. M^#^ denotes oxidized methionine.

^b^Phosphorylation sites were assigned based on MS/MS product ions. Parentheses indicate cases where an alternative site is possible.

**Table 3:** Tissue expression and subcellular location for proteins from our panel. This information was compiled from Human Protein Atlas (HPA) and from Ingenuity Pathway Analysis.

No.	Database code	Entry name	Protein name	Tissue expression in the lung (from HPA; March 2012)			Subcellular location (from Ingenuity; March 2012)
				Pneumocytes	Macrophages	Other tissues
1	P08670	VIME_HUMAN	Vimentin	Y (strong)	Y (strong)	Y	Cytoplasm
2	P02794	FRIH_HUMAN	Ferritin heavy chain	N	Y (strong)	Y	Cytoplasm
3	P30086	PEBP1_HUMAN	Phosphatidylethanolamine-binding protein 1	Y (weak)	Y (weak)	Y	Cytoplasm
4	P13796	PLSL_HUMAN	Plastin-2	N	Y (strong)	Y (limited)	Cytoplasm
5	Q9H3Z4	DNJC5_HUMAN	DnaJ homolog subfamily C member 5	Y (moderate)	Y (moderate)	Y	Plasma membrane
6	P27816	MAP4_HUMAN	Microtubule-associated protein 4 (MAP 4)	Y (moderate)	Y (weak)	Y	Cytoplasm
7	P21333	FLNA_HUMAN	Filamin-A	Y (weak)	Y (strong)	Y	Cytoplasm
8	P08575	PTPRC_HUMAN	Receptor-type tyrosine-protein phosphatase C	N	Y (weak)	Y (limited)	Plasma membrane
9	P01042	KNG1_HUMAN	Kininogen-1	N	N	Y (distinct in plasma; other limited)	Extracellular space
10	P02765	FETUA_HUMAN	Alpha-2-HS-glycoprotein	N	Y (moderate)	Y (plasma; other limited)	Extracellular space
11	Q15637	SF01_HUMAN	Splicing factor 1	Y (moderate)	Y (moderate)	Y	Nucleus
12	Q9UK76	HN1_HUMAN	Hematological and neurological expressed 1 protein				Nucleus
13	Q7Z3D4	LYSM3_HUMAN	LysM and putative peptidoglycan-binding domain-containing protein 3	Y (moderate)	Y (strong)	Y	unknown
14	P02671	FIBA_HUMAN	Fibrinogen alpha chain	N	Y (moderate)	Y (limited)	Extracellular space
15	P09651	ROA1_HUMAN	Heterogeneous nuclear ribonucleoprotein A1	Y (strong)	Y (strong)	Y	Nucleus
16	P51858	HDGF_HUMAN	Hepatoma-derived growth factor;	Y (strong)	Y (moderate)	Y	Extracellular space
17	Q9H2C0	GAN_HUMAN	Gigaxonin	N	Y (moderate)	Y	Cytoplasm
18	Q13637	RAB32_HUMAN	Ras-related protein Rab-32	Y (moderate)	Y (weak)	Y	Cytoplasm
19	P35579	MYH9_HUMAN	Myosin-9	Y (strong)	Y (moderate)	Y	Cytoplasm
20	P07900	HS90A_HUMAN	Heat shock protein HSP 90-alpha	N	N	Y (limited)	Cytoplasm
21	P08238	HS90B_HUMAN	Heat shock protein HSP 90-beta	Y (moderate)	Y (moderate)	Y	Cytoplasm
